# Mycophenolate pharmacokinetics and pharmacodynamics in belatacept treated renal allograft recipients – a pilot study

**DOI:** 10.1186/1479-5876-7-64

**Published:** 2009-07-27

**Authors:** Sara Bremer, Nils T Vethe, Helge Rootwelt, Pål F Jørgensen, Jean Stenstrøm, Hallvard Holdaas, Karsten Midtvedt, Stein Bergan

**Affiliations:** 1 Department of Medical Biochemistry, Rikshospitalet University Hospital, 0027 Oslo, Norway; 2 Institute of Clinical Biochemistry, University of Oslo, 0027 Oslo, Norway; 3 Section for Transplant Surgery, Rikshospitalet University Hospital, Oslo, 0027 Oslo, Norway; 4 Department of Medicine, Rikshospitalet University Hospital, 0027 Oslo, Norway; 5 School of Pharmacy, University of Oslo, 0316 Oslo, Norway

## Abstract

**Background:**

Mycophenolic acid (MPA) is widely used as part of immunosuppressive regimens following allograft transplantation. The large pharmacokinetic (PK) and pharmacodynamic (PD) variability and narrow therapeutic range of MPA provide a potential for therapeutic drug monitoring. The objective of this pilot study was to investigate the MPA PK and PD relation in combination with belatacept (2^nd ^generation CTLA4-Ig) or cyclosporine (CsA).

**Methods:**

Seven renal allograft recipients were randomized to either belatacept (n = 4) or cyclosporine (n = 3) based immunosuppression. Samples for MPA PK and PD evaluations were collected predose and at 1, 2 and 13 weeks posttransplant. Plasma concentrations of MPA were determined by HPLC-UV. Activity of inosine monophosphate dehydrogenase (IMPDH) and the expressions of two *IMPDH *isoforms were measured in CD4+ cells by HPLC-UV and real-time reverse-transcription PCR, respectively. Subsets of T cells were characterized by flow cytometry.

**Results:**

The MPA exposure tended to be higher among belatacept patients than in CsA patients at week 1 (P = 0.057). Further, MPA concentrations (AUC_0–9 h _and C_0_) increased with time in both groups and were higher at week 13 than at week 2 (P = 0.031, n = 6). In contrast to the postdose reductions of IMPDH activity observed early posttransplant, IMPDH activity within both treatment groups was elevated throughout the dosing interval at week 13. Transient postdose increments were also observed for *IMPDH1 *expression, starting at week 1. Higher MPA exposure was associated with larger elevations of *IMPDH1 *(r = 0.81, P = 0.023, n = 7 for MPA and *IMPDH1 *AUC_0–9 h _at week 1). The maximum *IMPDH1 *expression was 52 (13–177)% higher at week 13 compared to week 1 (P = 0.031, n = 6). One patient showed lower MPA exposure with time and did neither display elevations of IMPDH activity nor *IMPDH1 *expression. No difference was observed in T cell subsets between treatment groups.

**Conclusion:**

The significant influence of MPA on *IMPDH1 *expression, possibly mediated through reduced guanine nucleotide levels, could explain the elevations of IMPDH activity within dosing intervals at week 13. The present regulation of IMPDH in CD4+ cells should be considered when interpreting measurements of IMPDH inhibition.

## Background

Mycophenolic acid (MPA) is widely used in immunosuppressive regimens, combined with calcineurin inhibitors (CNI), corticosteroids, and frequently also induction therapy, to prevent allograft rejection after transplantation. Currently, two MPA formulations are available, the prodrug ester mycophenolate mofetil (MMF) and the enteric-coated mycophenolate sodium.

Inosine monophosphate dehydrogenase (IMPDH) catalyzes the rate-limiting step of *de novo *guanine nucleotide synthesis. The enzyme activity is constituted by two isoenzymes, encoded by *IMPDH1 *and *IMPDH2*, which have similar kinetic properties and share 84% identity at the amino acid level [1]. However, the regulation and expression of the isoenzymes differ, and gene knockout models indicate distinct functions of IMPDH 1 and 2 [2,3]. Lymphocyte activation is associated with elevation of both isoenzymes, while neoplastic cells display marked up-regulation of *IMPDH2 *[4,5]. MPA exerts its immunosuppressive action by inhibiting IMPDH, and thereby the proliferation of activated lymphocytes [6].

MPA demonstrates a narrow therapeutic range and substantial inter- and intraindividual variability of pharmacokinetic (PK) and pharmacodynamic (PD) parameters. Renal function, albumin levels, concomitant medications and genetic polymorphisms of transporters and UDP-glucuronosyltransferases are among factors that influence MPA PK profiles [7,8]. Furthermore, MPA exposure is reported to increase over time after transplantation [9]. The activity of IMPDH, representing a PD marker, depends on cell type and cycle status and probably also concomitant medication and genetic variants of the *IMPDH *genes [4,10,11]. Despite the variability of MPA PK and PD, most immunosuppressive protocols prescribe fixed doses ranging from 0.75 to 1.5 g MMF twice a day.

Several strategies have been suggested to individualize MPA therapy and improve the clinical outcome after transplantation. The area under the MPA concentration versus time curve (AUC) from 0 to 12 hours correlates with clinical outcome after transplantation but is impractical for routine monitoring, and various limited sampling schemes have been evaluated [12-14]. Measurement of IMPDH activity may provide a more direct estimation of drug efficacy, and is investigated as a PD approach for individualization of MPA therapy [15,16]. Long-term MPA treatment has been associated with induced IMPDH activity and expression [10,17-20]. However, the results are conflicting and depend on the investigated cell populations and methodology. Furthermore, concomitant medications (*e.g*. high doses of corticosteroids) and the transplantation surgery itself may influence the activity and expression of IMPDH [10]. The clinical implications of these findings remain to be elucidated and further characterization of the IMPDH isoenzymes during MPA exposure is needed in the process of establishing strategies for PD based monitoring of MPA.

The introduction of CNIs resulted in dramatic improvements in short-term outcome after transplantation. However, long-term CNI use is associated with nephrotoxicity and cardiovascular morbidities that may increase the risk of late allograft loss and death. Belatacept, a second generation cytotoxic T-lymphocyte antigen-4 (CTLA4)-Ig fusion protein, is investigated as an alternative to CNIs following transplantation. It binds with high affinity to CD80 and CD86, thereby resulting in T cell anergy and apoptosis [21]. A phase 2 trial in renal allograft recipients (n = 218) reports similar efficacy, higher glomerular filtration rates and less frequent chronic allograft nephropathy with belatacept compared to cyclosporine (CsA) [22].

Several studies have demonstrated a PK interaction between CsA and MPA, resulting in lower MPA exposure [23,24]. Data on PK and PD of MPA in combination with belatacept are limited. The present investigation is a supplemental study appended to the BENEFIT-EXT phase 3 trial in transplant patients receiving grafts from extended criteria donors (BMS protocol IM103027) [25]. This is an observational, pilot study in renal transplant patients receiving MMF in combination with either belatacept or CsA. The objective was to investigate the relation between PD and PK characteristics of MPA in the two treatment groups during the early posttransplantation period. Measurements of MPA concentrations were used for PK evaluations, while PD investigations involved determination of IMPDH activity, analyses of *IMPDH 1 *and *2 *expression and characterization of T cell subpopulations. The PK and PD profiles of MPA changed with time after transplantation.

## Materials and methods

### Study subjects

From October 2006 to February 2007, seven adult patients receiving grafts from extended criteria donors were included in the BENEFIT-EXT study at Rikshospitalet University Hospital. Extended criteria donors were defined as donor age above 60 years, donor age above 50 years and other donor co-morbidities, cold ischemia time above 24 hours or donation after cardiac death. The inclusion and exclusion criteria are described in detail in the BENEFIT-EXT study protocol [25]. Biopsies were performed in cases of suspected rejection (Banff '97 grading system) [26]. Demographic and clinical data were collected from medical records.

Patients were randomized into three arms with CsA in one arm and belatacept (less intensive or more intensive, respectively) in the two others. Within the study period, both belatacept regimens included doses of 10 mg/kg administered as a 30 minutes intravenous (iv) infusion. Doses were given at day 1 and 5, and at weeks 2, 4, 8 and 12 for both regimens. The more intensive regimen included additional doses at weeks 6 and 10 [25]. Additional immunosuppression consisted of MMF (CellCept^®^, Roche, Basel, Switzerland) 1 g twice daily, corticosteroids and induction therapy with basiliximab (Simulect^®^, Novartis, Basel, Switzerland) 20 mg on day 0 (transplantation day) and day 4. Corticosteroids were given as iv methylprednisolone, 540 mg on day 0 and 250 mg on day 1, followed by per oral prednisolone starting at 100 mg/day, tapered by 10 mg/day and maintained at 20 mg/day the first month, at 15 mg/day the second month and at 10 mg/day the third month. CsA was dosed according to protocol to reach target whole blood through concentrations (C_0_) of 150–300 μg/L the first month posttransplant, and then lowered to 100–250 μg/L. All patients received prophylactic antiviral therapy consisting of valganciclovir or valaciclovir.

The protocols of both the BENEFIT-EXT trial and the present sub-study were approved by the regional committee for medical research ethics. The BENEFIT-EXT protocol was also approved by the Norwegian Medicines Agency. Written informed consent was obtained from all participants.

### Samples

Samples were collected on one occasion before transplantation and for 9 hour-profiles at approximately 1, 2 and 13 weeks posttransplant (referred to as week 1, 2 and 13). The PK-PD profiles were abbreviated to 0 to 9 hours postdose for practical reasons. Samples for 9 hour-profiles were drawn after an overnight fast before administration of the morning dose of immunosuppression, and at 0.5, 1, 1.5, 2, 3, 4, 5, 6 and 9 hours postdose. *IMPDH *expressions were not determined at 0.5 and 1.5 hours. Cell subsets were characterized in the predose and 2 hours postdose samples only. At each time point 10 mL whole blood was collected in EDTA tubes. Samples were immediately processed for CD4+ cell isolation, separation of plasma and staining of cells for flow cytometric characterization.

Enzyme activity and gene expression measurements were performed in CD4+ cells. These cells are relevant considering their role in allograft rejection as well as being among the target cells for the action of MPA. The cells were isolated from whole blood within an hour after sampling by the use of paramagnetic beads with antibodies against CD4 (Dynabeads^® ^CD4, Invitrogen, Carlsbad, CA) as described in detail elsewhere [27,28]. Analyses of biochemical and haematological parameters were performed according to standard methods at the clinical laboratory.

To evaluate the variability of IMPDH activity and gene expression without influence of medication or exposure to alloantigens, CD4+ cells from healthy individuals (n = 5) were investigated. Samples were drawn every 2 hours over 6 hour intervals starting at 8 AM as described in detail elsewhere [16,29].

### Concentrations of immunosuppressive drugs

Total plasma concentrations of MPA were measured by high-performance liquid chromatography assay with UV-detection (HPLC-UV) [30]. Routine measurement of whole blood CsA C_0 _was performed by the CEDIA^® ^immunoassay (Microgenics corp., Fremont, CA) on a Modular analytics instrument (Roche Diagnostics, Mannheim, Germany).

### Enzyme activity

For the quantification of IMPDH activity in CD4+ cells, intracellular MPA concentrations were restored by incubating the isolated cells in filtrated plasma originating from the same sample. The IMPDH activity was determined in cell lysates using an HPLC-UV assay for determination of xanthine derived from xanthosine monophosphate (XMP) [27]. Activities were expressed as the XMP production rate (pmol XMP per 1.0 × 10^6 ^CD4+ cells per min). For each dosing interval, predose (A_0_), maximum (A_max_), minimum (A_min_) and AUC enzyme activities were determined.

### Gene expression

The gene expressions of *IMPDH 1 *and *2 *in CD4+ cells were quantified by a validated reverse transcription-PCR method on a LightCycler^® ^480 instrument (Roche Applied Science) as previously described [28]. Briefly, total RNA was extracted and reverse transcribed using random primers. Sequences of *IMPDH1 *and *IMPDH2*, and the reference genes aminolevulinate delta-synthase1, β2-microglobulin and ribosomal protein L13A, were amplified in separate reactions including hybridization probes for specific real-time product detection. Crossing points were defined by the second derivative maximum method and target gene expressions were calculated relative to the geometric mean expression of the reference genes. Based on the dose interval samples, predose (E_0_), maximum (E_max_), minimum (E_min_) and AUCs for *IMPDH1 *and *2 *gene expressions were calculated for each profile.

### Quantification of T cell subsets

The numbers of total T cells (CD3+), as well as subpopulations of helper (CD4+) and cytotoxic (CD8+) T cells were determined by flow cytometry. These subsets were further characterized based on the expression of CD45RA and CD45RO isoforms indicating naïve and antigen experienced (activated/memory) lymphocytes, respectively.

Absolute quantification of T cell subsets was performed using TruCount tubes according to the manufacturer's instructions. Briefly, 50 μL EDTA blood was added to tubes containing a given number of beads and cells were stained with titrated amounts of anti-CD3-PerCP, anti-CD45 RO-PE, anti-CD45 RA-APC and anti-CD4-FITC or anti-CD8-FITC monoclonal antibodies (mAb). Isotype-matched control anti-mouse mAb and non-labeled cells were included for each sample. Erythrocytes were lysed by adding 450 μL FACS Lysing Solution. The tubes and all reagents were supplied by BD (Becton Dickinson Biosciences, Oxford, UK). Flow cytometric analyses were performed within 24 hours after labeling on a FACSCalibur (BD) flow cytometer using the CellQuest Software (BD) for data acquisition. The bead population and CD3+ cell versus side scatter population were manually gated.

### Data analysis and statistics

Results of the RT-PCR assays were analyzed using the LightCycler 480 Software v.1.5 (Roche Applied Science). All gene expression measurements were performed in triplicate. Absolute cell counts were calculated by the CellQuest Software based on the gated bead population.

Postdose data of gene expression and enzyme activity were normalized to individual predose levels. Based on the steady-state of MMF dosing, AUCs were calculated by the linear trapezoid method for intervals 0–6 hours, 0–9 hours and 4–9 hours as indicated (AUC_0–6 h_, AUC_0–9 h_, AUC_4–9 h_, respectively). All results are presented as median (range) unless otherwise specified.

Statistical tests were performed using SPSS statistical software version 16.0 (SPSS Inc., Chicago, IL). The Mann-Whitney test was used for comparisons of unpaired data, while the Wilcoxon signed rank test was used for paired data. Pearson's *r *was used for correlation analyses. Statistical significance was considered at P < 0.05 (two-tailed).

## Results

### Patient population

The planned enrolment for the BENEFIT-EXT trial at Rikshospitalet University Hospital was 12 patients. However, only 7 patients receiving allografts from extended criteria donors were recruited at our center within the inclusion period. Out of these, 3 patients were randomized to receive CsA, while 4 patients received belatacept regimens.

Baseline characteristics are summarized in Table 1. There were no significant demographic differences between the treatment groups. One of the belatacept patients withdrew from the study after the 6 hours postdose sampling at week 2. Data from this profile were omitted from the AUC calculations.

**Table 1:** Patient characteristics

	Belatacept (n = 4)		CsA (n = 3)	
Age, years	74	(68–78)	66	(29–71)

Gender, M/F	3/1		3/0	

Bodyweight, kg	63.1	(58.7–85.6)	92.3	(75.7–96.0)

Body mass index, kg/m^2^	22.9	(18.6–28.0)	26.7	(23.1–26.9)

Donor, DD/LD	4/0		3/0	

Previous transplants	0		0	

Dialysis pretransplant	3		1	

Observation day after transplantation (day 0)				

Week 1	7	(6–8)	6	(6–7)

Week 2	14.5	(13–15)	16	(14–20)

Week 13	90.5	(78–95)	91	(77–93)

Number of HLA mismatches				

Total	2.5	(2–3)	1	(0–3)

DR	0.5	(0–1)	1	(0–1)

Duration of cold ischemia (h)	16.5	(9.2–23.6)	13.4	(12.7–15.1)

CMV serostatus				

D+/R+	4		1	

D+/R-	0		2	

CMV, cytomegalovirus; D, donor; DD, deceased donor; LD, living donor; R, recipient

No cytomegalovirus breakthrough disease was identified during the study period. Biopsy verified acute rejection, graft loss and death were absent during the 13 weeks follow-up. Renal function improved significantly the first weeks after transplantation. Plasma concentrations of albumin, total bilirubin, and ALAT were stable throughout the study period.

### MPA pharmacokinetics

**Figure 1:**
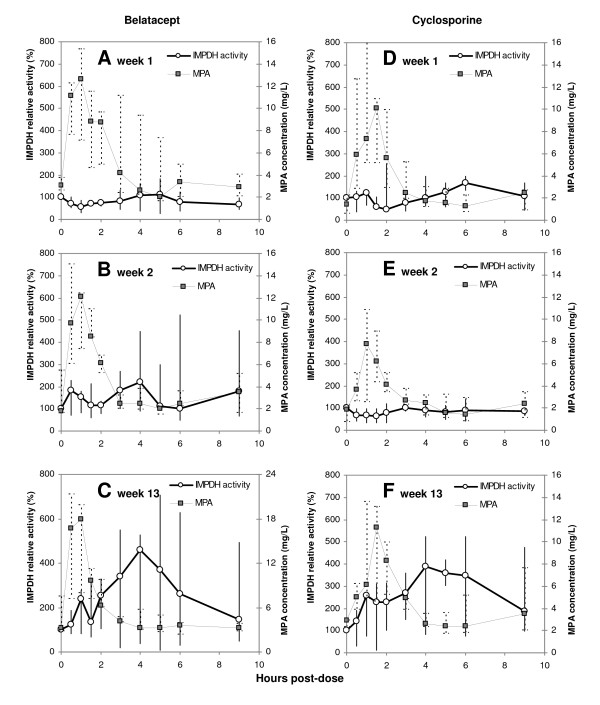
**Median inosine monophosphate dehydrogenase (IMPDH) activity (% of predose) and mycophenolic acid (MPA) concentrations among renal allograft recipients**. The vertical lines represent the range of total observations. Profiles of patients in the belatacept group (n = 3) at weeks 1, 2 and 13 (A, B and C) and the cyclosporine group (n = 3) at weeks 1, 2 and 13 (D, E and F). (Observe scale on right y-axis of C.)

Two patients, both in the belatacept arm, had their MMF dosing reduced to 1.5 g/day between weeks 2 and 13, both due to drops in leukocyte count. Steady-state conditions with respect to MPA were established in both patients before the investigations at week 13. The other patients remained on MMF doses of 1 g twice a day throughout the follow-up. Pharmacokinetic data of MPA are summarized in Table 2 and concentration profiles are depicted in Figure 1. The interindividual variability in MPA concentration was substantial and highest early posttransplant. Within the whole group, up to 4- and 7-fold differences were observed for MPA C_0 _(week 2) and AUC_0–9 h _(week 1), respectively. The first week posttransplant, MPA C_0 _seemed to be higher among belatacept patients (P = 0.057, n = 4 and n = 3) and 3 of 4 belatacept patients demonstrated higher MPA AUC_0–9 h _than the CsA patients.

**Table 2:** MPA exposure and IMPDH activity

		Treatment group				Total	
*MPA plasma concentration*	Week	Belatacept (n = 4)		Cyclosporine (n = 3)			

C_0 _(mg/L)	1	3.1	(2.7–3.8)	1.4	(0.7–2.3)	2.7	(0.7–3.8)
	
	2	1.9	(1.7–5.5)	1.9	(0.8–2.3)	1.9	(0.8–5.5)
	
	13	3.2	(2.9–7.6)	2.9	(2.4–3.0)	3.0	(2.4–7.6)

AUC_0–9 h_ (mg × h/L)	1	44.4	(28.2–70.8)	37.1	(17.9–40.1)	40.1	(17.9–70.8)
	
	2	35.1	(33.6–47.6)	26.4	(16.3–37.8)	34.4	(16.3–47.6)
	
	13	48.5	(39.1–64.1)	37.4	(27.2–59.0)	43.8	(27.2–64.1)

C_max _(mg/L)	1	12.8	(7.7–15.4)	11.0	(5.2–19.5)	11.3	(5.2–19.5)
	
	2	12.1	(9.7–15.1)	7.8	(4.4–10.9)	10.9	(4.4–15.1)
	
	13	17.9	(8.1–21.4)	11.3	(5.3–13.7)	12.5	(5.3–21.4)

							

*IMPDH activity in CD4+ cells*							

A_0_ (pmol/10^6 ^cells/min)	0	0.24	(0.16–0.31)	0.61	(0.3–0.95)	0.31	(0.16–0.95)
	
	1	0.96	(0.70–1.4)	0.63	(0.37–1.53)	0.92	(0.37–1.53)
	
	2	0.43	(0.25–0.71)	1.1	(0.66–1.53)	0.60	(0.25–1.53)
	
	13	0.70	(0.32–2.7)	0.28	(0.2–1.87)	0.51	(0.2–2.72)

AUC_0–9 h_ (% of A_0 _× h)	1	760	(472–908)	1197	(904–1491)	884	(472–1491)
	
	2	1168	(694–3142)	760	(488–1032)	1032	(488–3142)
	
	13	3034	(414–3784)	3044	(765–3111)	3039	(414–3784)

A_min_ (% of A_0_)	1	45.5	(25.4–58.1)	46.1	(39.0–100)	46.1	(25.4–100)
	
	2	77.4	(48.0–100)	64.3	(32.6–96.0)	77.4	(32.6–100)
	
	13	100	(7.6–100)	100	(13.0–100)	100	(7.6–100)

A_max_ (% of A_0_)	1	141	(103–184)	170	(100–254)	160	(100–254)
	
	2	255	(113–524)	119	(100–137)	184	(100–524)
	
	13	627	(106–707)	523	(148–525)	524	(106–707)

Data are given as median (range). The belatacept group includes 3 patients at week 13 and for the maximum, minimum and AUC calculations at week 2. A_0_, predose activity; A_max_, maximum activity; A_min_, minimum activity; AUC, area under the variable versus time curve; C_0_, predose concentration, C_max_, maximum concentration; C_min_, minimum concentration, IMPDH, inosine monophosphate dehydrogenase; MPA, mycophenolic acid.

The maximum plasma concentrations (C_max_) of MPA appeared 1 (0.5–2) hour postdose. Following C_max_, secondary MPA concentration peaks were observed 5 (2–9) hours postdose and were more pronounced for belatacept patients than for CsA patients. Limited MPA concentration profiles were calculated from 4 to 9 hours to estimate potential impact of enterohepatic circulation. The MPA AUC_4–9 h _was numerically higher among belatacept patients than for CsA patients at week 1, being 15.2 (10.4–27.1) mg × h/L and 7.8 (6.2–13.3) mg × h/L, respectively (P = 0.114, n = 4 and n = 3).

Doses of CsA were tapered according to CsA C_0 _measurements and were median 550 (450–825) mg, 550 (400–575) mg and 300 (300–350) mg at week 1, 2 and 13, respectively. The corresponding CsA C_0 _were median 190 (160–380) μg/L, 265 (180–295) μg/L and 175 (140–180) μg/L. The reduction of CsA exposure was accompanied by increasing MPA concentrations. The association between MPA C_0 _and CsA C_0_, as well as CsA dose, displayed correlation coefficients (r) of -0.74 (P = 0.023, n = 9; pooled CsA data) and -0.79 (P = 0.012, n = 9), respectively.

Considering the entire study population, the lowest MPA exposure was observed at week 2 and then increased with time. At week 13, MPA C_0 _was 60 (26–200)% higher (P = 0.031, n = 6), while MPA AUC_0–9 h _was 43 (11–67)% higher (P = 0.031, n = 6) compared to week 2. The elevation seemed to be most pronounced in CsA patients, although no significant difference was detected between groups (Table 2).

At week 1, MPA exposure was inversely correlated to bodyweight, with correlation coefficients of -0.90 (P = 0.005, n = 7) and -0.80 (P = 0.031, n = 7) for MPA C_0 _and AUC_0–9 h_, respectively. However, no significant relation was detected at later observations. Adjusted for bodyweight normalized doses, patients with belatacept displayed numerically higher MPA C_0_, 0.22 (0.18–0.23; n = 4) mg/L per mg/kg, than CsA patients, 0.13 (0.07–0.17; n = 3) mg/L per mg/kg, at week 1 (P = 0.057). The MPA exposure did not seem to be associated with plasma albumin, ALAT or bilirubin.

### Enzyme activity

Summarized data of IMPDH activity are presented in Figure 1 and Table 2. Pretransplant activity was variable and tended to be higher among CsA patients compared to belatacept patients. Following transplantation, predose activities (A_0_) seemed to be influenced by the present MPA C_0_, and no consistent trends were observed for A_0 _versus time since transplantation (Table 2).

**Figure 2:**
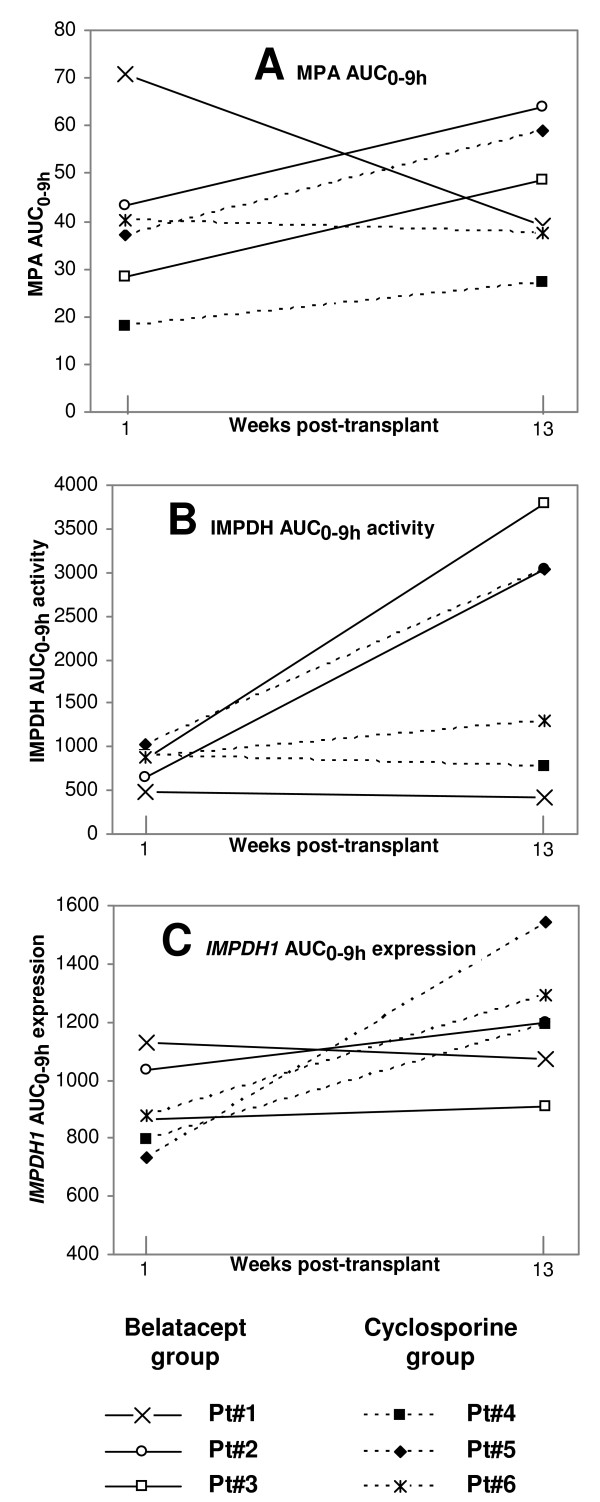
**Individual 0–9 hours area under the curve (AUC) for 6 renal transplant patients at week 13 compared to week 1**. Solid lines denote belatacept patients (n = 3) while broken lines represent CsA patients (n = 3). Data are provided for A: mycophenolic acid (MPA) AUC_0–9 h_, B: inosine monophosphate dehydrogenase (IMPDH) activity AUC_0–9 h _and C: *IMPDH1 *expression AUC_0–9 h_.

The postdose activities of IMPDH were strongly influenced by MPA exposure. At week 1, the activity profiles for 6 of the patients were inversely related to MPA concentrations with maximum 57 (42–75)% enzyme inhibition around MPA C_max _(Figure 1). The AUC_0–9 h _activities displayed inverse correlations to MPA C_0 _(r = -0.91, P = 0.012, n = 6) and MPA C_max _(r = -0.86, P = 0.028, n = 6), implying greater inhibition of IMPDH with higher MPA exposure. However, this relation changed with time posttransplant. At week 13, IMPDH activity increased postdose within both treatment groups, reaching up to 7-times A_0 _before returning towards predose activities (Figure 1). Considering AUC_0–9 h _activity, 4 of 6 patients demonstrated substantial increases reaching 3.6 times the activity of week 1 (Figure 2). Compared to week 2, the AUC_0–9 h _activity was 81 (25–322)% higher at week 13 (P = 0.063, n = 5). Higher MPA C_max _was associated with increasing IMPDH activity, expressed as AUC_0–9 h _(r = 0.80, P = 0.058, n = 6) and A_max _(r = 0.88, P = 0.051, n = 6). Compared to healthy controls (n = 5), the CsA treated patients (n = 3) showed higher IMPDH AUC_0–6 h _activity at week 13 (P = 0.036). Within the belatacept group, 2 of 3 patients displayed higher activity than the controls (Additional file 1: IMPDH activity and IMPDH1 expression in patients on MMF therapy compared to healthy individuals).

### Gene expression

The pretransplant expression of *IMPDH2 *was 2.1 (1.6–2.7) times higher than *IMPDH1 *in CD4+ cells. Predose expressions (E_0_) of *IMPDH 1 *and *2 *were highest and most variable the first week posttransplant, being 104 (20–150) % and 18.8 (7.2–75) % above the levels at week 13, respectively (P = 0.031, n = 6 for both). Predose expressions were comparable at week 2 and 13 (Table 3).

**Table 3:** *IMPDH1 *expression

		Treatment group				Total	
*IMPDH1*	Week	Belatacept (n = 4)		Cyclosporine (n = 3)			

E_0_	0	0.63	(0.54–0.76)	0.44	(0.37–0.79)	0.59	(0.37–0.79)
	
	1	0.56	(0.32–1.1)	0.75	(0.67–0.75)	0.67	(0.32–1.1)
	
	2	0.45	(0.17–0.54)	0.54	(0.43–0.62)	0.50	(0.17–0.62)
	
	13	0.42	(0.25–0.59)	0.31	(0.30–0.43)	0.36	(0.25–0.59)

AUC_0–9 h_ (% of E_0 _× h)	1	1018	(866–1128)	794	(736–881)	880	(736–1128)
	
	2	1146	(781–1278)	784	(741–1146)	1145	(741–1622)
	
	13	1070	(911–1201)	1291	(1193–1540)	1197	(911–1540)

E_min_ (% of E_0_)	1	85.3	(75.3–115)	69.3	(46.8–92.2)	82.0	(46.8–115)
	
	2	94.4	(80.2–103)	71.1	(60.7–94.3)	87.3	(60.7–103)
	
	13	97.0	(57.2–99.6)	113	(89.5–117)	98.3	(57.2–117)

E_max _(% of E_0_)	1	140	(108–143)	105	(102–122)	121	(102–143)
	
	2	147	(105–189)	107	(104–151)	127	(104–189)
	
	13	161	(133–196)	203	(173–222)	185	(133–222)

Data are given as median (range). The belatacept group includes 3 patients at week 13 and for the maximum, minimum and AUC calculations at week 2. E_0_, predose expression; E_max_, maximum expression; E_min_, minimum expression; AUC, area under the variable versus time curve.

**Figure 3:**
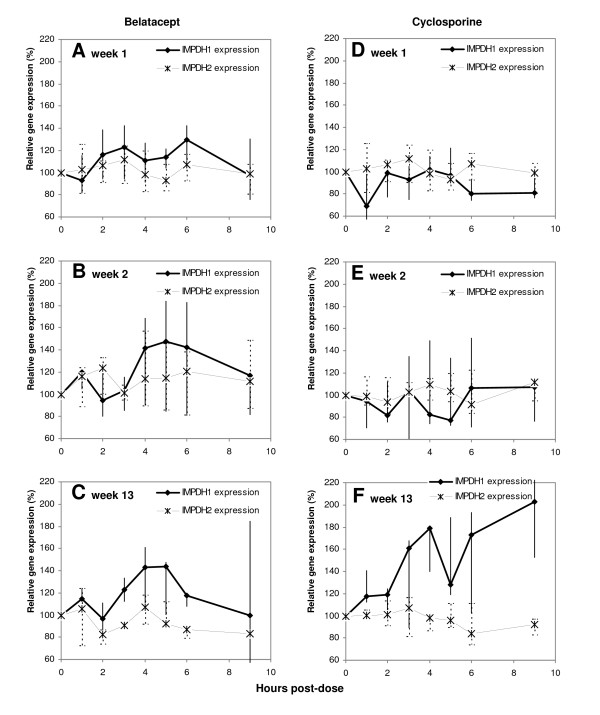
**Median gene expressions of *IMPDH1 *and *IMPDH2 *(% of predose) among renal allograft recipients**. The vertical lines correspond to the range of total observations. Profiles of patients in the belatacept group (n = 3) at weeks 1, 2 and 13 (A, B and C) and the cyclosporine group (n = 3) at weeks 1, 2 and 13 (D, E and F).

The 9 hour-profiles showed rapid changes of *IMPDH1 *expression postdose, while *IMPDH2 *expression was relatively stable (Figure 3). At week 1, *IMPDH1 *expression was transiently upregulated for belatacept patients, while CsA patients displayed downregulation. With longer time on immunosuppressive therapy, including higher MPA exposure, increasing transient inductions of *IMPDH1 *expression were observed postdose for both treatment groups (Table 3). At week 13, the maximum expression (E_max_, % of E_0_) of *IMPDH1 *was 52 (13–177)% higher than at week 1 (n = 6, P = 0.031). A similar trend was observed for *IMPDH1 *AUC_0–9 h _expression (n = 6, P = 0.094). Compared to healthy controls (n = 5), the patients (n = 6) demonstrated higher *IMDPH1 *E_max _at week 13 (P = 0.004), being 101 (100–116)% and 167 (118–193)%, respectively. Considering *IMPDH1 *AUC_0–6 h_expression, CsA patients (n = 3) displayed higher levels at week 13 than controls (P = 0.036). Among belatacept patients (n = 3), *IMPDH1 *AUC_0–6 h _expression was elevated at week 1 (P = 0.032) and tended to be increased at week 13 (P = 0.071), compared to healthy controls (Additional file 1: IMPDH activity and *IMPDH1 *expression in patients on MMF therapy compared to healthy individuals). One of the patients with MMF dose reduction experienced lower MPA exposure with time, and did neither display elevations of IMPDH activity nor *IMPDH1 *expression (Figure 2). The first week posttransplant, *IMPDH1 *AUC_0–9 h _expression correlated with MPA C_0 _(r = 0.76, P = 0.047, n = 7) and MPA AUC_0–9 h _(r = 0.81, P = 0.027, n = 7). An association was also observed between minimum *IMPDH1 *expression (E_min_) and MPA AUC_0–9 h _(r = 0.82, P = 0.023, n = 7). This implies that higher MPA exposure is associated with larger increases of *IMPDH1 *expression postdose.

The *IMPDH1 *isoform demonstrated stronger correlations to IMPDH activity than *IMPDH2*. At week 1, there was an inverse correlation of -0.88 (P = 0.02, n = 6) between *IMPDH1 *E_max _and IMPDH A_max _indicating that lower IMPDH activity was accompanied by larger elevations of *IMPDH1 *expression. This relation changed with time, and 13 weeks posttransplant *IMPDH1 *AUC_0–9 h _expression displayed positive correlations with IMPDH AUC_0–9 h _activity (r = 0.94, P = 0.005, n = 6) and A_max _(r = 0.90, P = 0.038, n = 5). Although *IMPDH2 *was the dominant isoform predose, the ratio of *IMPDH2 *to *IMPDH1 *expression declined after dosing toward ratios of about 1 for some patients.

No significant associations were observed between activity or gene expressions of IMPDH and age, time since transplantation, dialysis, infections or HLA-DR mismatches.

### T cell subsets

Characterization of T cell subsets was only performed in 6 of the 7 patients, for technical reasons.

Before transplantation, patients demonstrated a wide range of T cell counts, with up to 2.2- and 2.8-fold variation for both CD4+ and CD8+ cells. Following transplantation, the number of both subpopulations tended to decrease among belatacept patients while the T cell profiles for CsA patients were more variable. At week 2, two of three CsA patients displayed up to 2-fold increases of CD4+ and CD8+ T cells, while reductions of 16.5 (7.7–49.5)% and 31.7 (32.0–49.6)% were observed for belatacept patients.

The proportions of naïve (CD45RA) and memory (CD45RO) T cells were comparable in both treatment groups, displaying CD45RA to CD45RO ratios of 0.61 (0.37–1.0) and 1.7 (1.1–3.0) for CD4+ and CD8+ cells (n = 6), respectively, before transplantation. The percentage of CD4+ cells with memory phenotype tended to decline posttransplant within both groups. At week 13, the proportion of memory CD4+ cells was 12.3 (3.5–22)% (P = 0.063, n = 6) lower than pretransplant.

The largest alteration in T cell subsets from pre- to post-dose, was observed for CD4+ cells at week 13 with reductions of 45.8 (24.6–52.8)% (n = 6, P = 0.063). However, the proportions of naïve and memory cells were comparable before and after dose.

## Discussion

This is the first study of MPA PK and PD relations among renal allograft recipients receiving belatacept compared to patients with CsA. Data from healthy individuals were included to account for possible diurnal or random variability of IMPDH.

Although standard MMF doses were applied, there was a considerable variability of MPA exposure among individuals. Early posttransplant, belatacept patients showed higher MPA concentrations, as well as more pronounced secondary concentration peaks, than CsA patients. Other comedication and parameters of renal and hepatic function were similar between the groups, and the inverse correlation between CsA and MPA concentrations suggest an effect of CsA on MPA exposure. Despite MMF dose reductions for two belatacept patients, the MPA exposure increased significantly from week 2 to week 13 when considering the whole population. The elevation might be related to the tapering of CsA and corticosteroid doses and improvement of renal function.

The PK of MPA is reported to be influenced by renal function, albumin levels and concomitant medications [31]. Genetic polymorphisms of transporters, *e.g*. multidrug resistance-associated protein 2 (MRP2), and UDP-glucuronosyltransferases may also contribute to variable MPA exposure [7,8]. Several studies have reported lower MPA concentrations when used in combination with CsA than used with tacrolimus, sirolimus or alone [23,24]. This is probably due to CsA mediated inhibition of MRP2, which is involved in enterohepatic circulation of MPA [32]. Furthermore, MPA exposure is reported to increase with time posttransplant. The mechanisms are multifactorial and may include changes in comedication, protein binding, renal function, liver disease and red blood cell counts [33,34].

In contrast to the inverse relation between MPA concentrations and IMPDH activity in CD4+ cells early posttransplant, prolonged MPA administration was associated with transient elevations of activity within dose intervals. This shifting IMPDH response is supported by the opposite correlations at week 1 and 13 between MPA exposure and IMPDH activity, and may provide an explanation for why higher concentrations of MPA do not result in markedly higher inhibition [16].

The regulation of the two IMPDH isoenzymes was further investigated by gene expression analysis. Following dosing, the expression of *IMPDH1 *displayed rapid and transient changes. Increasing MPA exposure was associated with larger inductions of *IMPDH1*. This might contribute to the associated elevation of IMPDH activity at week 13. The relative increase of *IMPDH1 *versus *IMPDH2 *expression supports marked contributions of IMPDH1 to the measured activity within dosing intervals.

The present changes of IMPDH activity and *IMPDH1 *expression in CD4+ cells are consistent with previous observations in mononuclear cells from transplant patients [20]. In addition, a study in healthy volunteers receiving different doses of MMF reported that regulation of *IMPDH1 *expression was associated with MPA exposure [29]. The *IMPDH1 *gene may be regulated through changes in guanine nucleotides, or potentially by direct effects of MPA. Previous reports suggest negative feedback regulation of IMPDH by guanine nucleotides in cultured human cells and in yeast [35,36]. In CD4+ cells from healthy individuals, low MPA exposure seemed to be associated with elevations of guanine nucleotides and subsequent reductions of *IMPDH1 *expression [16,29]. In contrast, higher and repeated MPA exposure may lead to depletion of intracellular guanine nucleotides and subsequent upregulation of *IMPDH1 *expression as was observed in the present study. Concomitant measurement of guanine nucleotides and gene expression in a larger cohort is necessary to confirm this hypothesis. Furthermore, potential effects of comedications like corticosteroids, basiliximab or the antiviral prophylaxis cannot be excluded.

Prolonged MPA administration has been associated with increased predose IMPDH activity in whole blood and erythrocytes but not lymphocytes [10,17-19]. The rapid and transient induction of IMPDH in CD4+ cells contrasts the gradual elevation in erythrocytes, which may originate from an induction in earlier differentiation stages that persists during erythrocyte maturation.

Traditionally, IMPDH1 has been regarded constitutive, while IMPDH2 was considered to be the inducible isoenzyme and primary target for immunosuppression [37]. More recent findings reveal that both isoenzymes are essential for lymphocyte proliferation and potentially important for immunosuppressive effects [4]. Furthermore, associations between genetic variants of *IMPDH1 *and a form of autosomal dominant retinitis pigmentosa have increased the interest in this isoform [38]. The current study emphasizes different genetic control of the isoenzymes in CD4+ cells. Although the detailed mechanisms are unknown, *IMPDH1 *is reported to be subject to complex regulation involving three promoters and various transcripts [39]. Because IMPDH2 is approximately 5 times more sensitive to MPA than IMPDH1 [40], a relative increase of IMPDH1 could have implications for the MPA effect.

Previous studies have described reduced CD4+ cell counts after initiation of immunosuppression [41]. This was also observed for the belatacept patients in the present study. In contrast, the increased CD4+ cell counts for two CsA patients at week 2 may be attributed to immune activation. Furthermore, the tendency towards reduced proportions of CD4+ memory cells within both treatment groups at week 13 may be explained by the current immunosuppression. It has generally been accepted that memory T cells do not require CD28-CD80/CD86 costimulation for recall responses. Recent studies have suggested that T cell costimulation is required for optimal IL-2 production and proliferation of both naïve and memory CD4+ T cells [42]. Despite having different mechanisms of action, both belatacept and CsA interfere with the IL-2 pathway, supporting the similar effects on T cell subsets. However, several exogenous (*e.g*. other immunosuppressants) and endogenous factors (*e.g*. circadian rhythm, stress) may also influence lymphocyte subsets and should be accounted for in further studies.

The isolation of variable numbers of CD4+ cells in each sample was compensated by relating IMPDH activity to cell counts and gene expressions to a reference gene index. However, various subsets of peripheral CD4+ T cells may display different levels of IMPDH activity and gene expressions. Alterations in these subsets could thereby influence the measured activity and gene expression. Although CD4+ cell counts changed, the proportions of naïve and memory cells remained stable after dose, indicating that IMPDH changes are not an effect of altered CD4+ cell populations.

The potential of a PD approach for MPA individualization has been supported by correlations between IMPDH levels and posttransplant outcomes. Sanquer *et al*. reported an up-regulation of predose *IMPDH1 *expression in mononuclear cells at acute rejection episodes [20]. Moreover, high pretransplant IMPDH activity in mononuclear cells and *IMPDH2 *expression in CD4+ cells have been associated with acute rejection episodes [10,15]. Recently, polymorphisms within the *IMPDH1 *and *IMPDH2 *genes have been suggested to impact baseline IMPDH activity and outcomes after transplantation [43,44]. Indeed, further investigations of IMPDH activity and regulation of the two isoenzymes are essential to elucidate the level of IMPDH inhibition that yields adequate immunosuppression. The present study suggests that MPA has a significant influence on *IMPDH1 *expression within the dose interval. This is an important aspect to consider when interpreting measurements of IMPDH inhibition.

The major limitation of this study is the low number of enrolled patients. This implies that the results should be interpreted with caution and that future prospective studies with larger cohorts are required to confirm the findings. The clinical outcome, including renal function, is investigated in detail in the ongoing BENEFIT-EXT trial [25].

## Conclusion

In the present pilot study, the IMPDH activity in CD4+ cells throughout dose intervals was significantly increased by week 13 compared to early posttransplant. This was observed both in cyclosporine and belatacept treated patients, and irrespective of higher MPA exposure. A marked increase of *IMPDH1 *expression within dose intervals, possibly mediated by reduced guanine nucleotide levels, may explain this paradox. The differences in MPA exposure between CsA and belatacept treated patients were as anticipated with reference to the documented CsA induced reductions in MPA exposure. No pronounced effects were observed of belatacept per se on MPA PK or PD.

## Competing interests

The authors declare that they have no competing interests.

## Authors' contributions

SB, StB, PFJ, HH, KM and JS participated in the design of the study. PFJ, HH, KM and JS provided the patients. The samples were collected by JS. SB, NTV, HR and StB contributed to the development of analytical methods. SB and NTV prepared the samples and performed sample and data analyzes. NTV, HR and StB helped to interpret data and draft the manuscript written by SB. All authors read and approved the manuscript.

SB, Sara Bremer; StB, Stein Bergan.

## Supplementary Material

Additional file 1 IMPDH activity and *IMPDH1 *expression in patients on MMF therapy compared to healthy individuals*. Data represent median (range) IMPDH activity and *IMPDH1 *expression in CD4+ cells from patients on MMF therapy (1, 2 and 13 weeks posttransplant) and healthy individuals. Click here for file
