# Supplementary material for: Mycophenolate pharmacokinetics and pharmacodynamics in belatacept treated renal allograft recipients – a pilot study
Source: J Transl Med. 2009 Jul 27;7:64. doi: 10.1186/1479-5876-7-64 (PMC2724496; doi:10.1186/1479-5876-7-64)
Supplement: Additional file 1 — IMPDH activity and IMPDH1 expression in patients on MMF therapy compared to healthy individuals*. Data represent median (range) IMPDH activity and IMPDH1 expression in CD4+ cells from patients on MMF therapy (1, 2 and 13 weeks posttransplant) and healthy individuals. [file 1479-5876-7-64-S1.doc]

**Additional file 1.** IMPDH activity and *IMPDH1* expression in patients on MMF therapy compared to healthy individuals*

|  |  |  | Patients on MMF therapy | | | | Healthy individuals | |
| --- | --- | --- | --- | --- | --- | --- | --- | --- |
|  |  | Week | Belatacept (n = 4)** | | Cyclosporine (n = 3) | | (n = 5) | |
| *IMPDH activity in CD4+ cells* |  |  |  | |  | |  | |
| AUC_0-6h_  (% of A_0_×h) |  | 1 | 760 | (472-908) | 1197 | (904-1491) | 691 | (636-758) |
|  |  | 2 | 1168 | (694-3142) | 760 | (488-1032) |  |  |
|  |  | 13 | 3034 | (414-3784) | 3044 | (765-3111) |  |  |
| A_max_  (% of A_0_) |  | 1 | 141 | (103-184) | 170 | (100-254) | 165 | (147-175) |
|  |  | 2 | 255 | (113-524) | 119 | (100-137) |  |  |
|  |  | 13 | 627 | (106-707) | 523 | (148-525) |  |  |
|  |  |  |  |  |  |  |  |  |
| *IMPDH1 expression in CD4+ cells* |  |  |  |  |  |  |  |  |
| AUC_0-6h_  (% of E_0_×h) |  | 1 | 705 | (574-709) | 597 | (538-600) | 554 | (482-697) |
|  |  | 2 | 788 | (523-1157) | 519 | (487-773) |  |  |
|  |  | 13 | 698 | (603-752) | 791 | (772-899) |  |  |
| E_max_  (% of E_0_) |  | 1 | 134 | (102-142) | 110 | (102-114) | 101 | (100-116) |
|  |  | 2 | 142 | (100-183) | 106 | (100-151) |  |  |
|  |  | 13 | 143 | (118-161) | 179 | (173-193) |  |  |

Data are given as median (range). *The results from healthy individuals are included for comparison and are recalculated for AUC_0-6h_ from data in a study by Vethe *et al*. and Bremer *et al.* (Vethe N.T. *et al*., *Ther Drug Monit* 2008, **30:** 647-655 and Bremer S. *et al*., *Int Immunopharmacol* 2009, **9:** 173-180). **The belatacept group includes 3 patients at week 2 and 13. Abbreviations: A_0_, predose activity; A_max_, maximum activity; AUC, area under the variable versus time curve; E_0_, predose expression; E_max_, maximum expression; IMPDH, inosine monophosphate dehydrogenase; MMF, mycophenolate mofetil.
